# Selenium and Selenoproteins at the Intersection of Type 2 Diabetes and Thyroid Pathophysiology

**DOI:** 10.3390/antiox11061188

**Published:** 2022-06-16

**Authors:** Francesca Gorini, Cristina Vassalle

**Affiliations:** 1 Institute of Clinical Physiology, National Research Council, 56124 Pisa, Italy; 2 Fondazione CNR-Regione Toscana Gabriele Monasterio, 56124 Pisa, Italy; cristina.vassalle@ftgm.it

**Keywords:** type 2 diabetes, insulin resistance, selenium, thyroid hormones, oxidative stress

## Abstract

Type 2 diabetes (T2D) is considered one of the largest global public-health concerns, affecting approximately more than 400 million individuals worldwide. The pathogenesis of T2D is very complex and, among the modifiable risk factors, selenium (Se) has recently emerged as a determinant of T2D pathogenesis and progression. Selenium is considered an essential element with antioxidant properties, and is incorporated into the selenoproteins involved in the antioxidant response. Furthermore, deiodinases, the enzymes responsible for homeostasis and for controlling the activity of thyroid hormones (THs), contain Se. Given the crucial action of oxidative stress in the onset of insulin resistance (IR) and T2D, and the close connection between THs and glucose metabolism, Se may be involved in these fundamental relationships; it may cover a dual role, both as a protective factor and as a risk factor of T2D, depending on its basal plasma concentration and the individual’s diet intake. In this review we discuss the current evidence (from experimental, observational and randomized clinical studies) on how Se is associated with the occurrence of T2D and its influence on the relationship between thyroid pathophysiology, IR and T2D.

## 1. Introduction

Diabetes is a serious and chronic condition characterized by high blood-glucose levels and is considered one of the largest global public-health concerns; it has a heavy impact on the quality of life of individuals and their families, as well as on social, financial and development issues [1,2]. According to the latest data from the Global Burden of Disease, in 2017, approximately 462 million individuals were affected by type 2 diabetes (T2D) at the global level, which equates to 6.28% of the world’s population, with the majority living in low-and middle-income countries [2,3]. The estimated global prevalence recorded a four-fold increase between 1980 and 2014 [4], and although incidence has started to decrease in some countries, the worldwide incidence of cases of T2D has increased by 104.6% from 1990 to 2017 [5,6]. Therefore, the number of people living with T2D, which accounts for more than 95% of total cases, is projected to rise by 25% in 2030 and 51% in 2045 if effective preventive interventions are not adopted [7,8]. In 2019, diabetes was the ninth leading cause of mortality, with an estimated 1.5 million deaths per year directly caused by T2D and 48% of all T2D-related deaths occurring before age 70 [2,8]. Importantly, half of the people with T2D are unaware of this condition [7].

T2D arises from pancreatic β-cell dysfunction and peripheral insulin resistance (IR) [9], and its pathogenesis, although very complex and yet to be fully elucidated, is mainly the result of modifiable risk factors [6,10]. Indeed, if the observed rise in prevalence is likely the result of longer survival because of early diagnosis and improved T2D treatment [7], the growing incidence of T2D in young people and adults is primarily due to obesity, increased consumption of unhealthy diets, and sedentary lifestyles; these are, overall, related to economic development [5]. Recent data indicate that imbalances in serum trace elements such as selenium (Se), magnesium, copper, chromium and zinc, may contribute to the pathogenesis and progression of T2D through the collapse of antioxidant defense [10,11]. In particular, a number of experimental and epidemiological studies have explored the controversial relationship between Se and T2D [12,13]. Selenium is an essential micronutrient for human health, as it is a structural amino acid (as selenocysteine, Sec) of both selenoproteins such as glutathione peroxidase 1 (GPx1)—a key antioxidant enzyme that protects cells from the harmful accumulation of hydrogen peroxide (H_2_O_2_) [14]—and thyroid deiodinases (DIOs), which are implicated in maintaining the homeostasis of thyroid hormones and their activity [15]. Thyroid diseases such as overt and subclinical hypothyroidism and autoimmune thyroiditis, have been linked to low Se status [16] but, due to the narrow range of adequate blood-Se concentrations, adverse effects to human health may occur below and above these thresholds [15]. Furthermore, abnormal levels of thyroid hormones (THs) have been associated with increased risk of T2D, although with mixed evidence [9,17]. Hence, in this review, we discussed both the role of Se in its relationship with T2D and, given the close connection between Se levels and thyroid pathophysiology, how this trace element may also influence the effects of THs on T2D.

References were identified by searching in Pubmed and Medline for original articles, reviews, systematic reviews and meta-analyses published in English up to 31 March 2022 useing the MeSH terms “diabetes”, “type 2 diabetes”, “T2D” and “insulin resistance” in combination with the term “selenium”. For the same period, an additional search strategy was also performed using the MeSH terms “thyroid”, thyroid hormones”, “hypothyroidism”, “hyperthyroidism” and “Graves’ disease” in combination with the terms “diabetes”, “type 2 diabetes”, “T2D” and “insulin resistance”, or in combination with the term “selenium”. The articles and reviews cited in these references were also reviewed.

## 2. Role of Oxidative Stress in Insulin Resistance and Type 2 Diabetes

The term “oxidative stress” defines any persistent disturbance in the balance between oxidants and antioxidants in favor of the oxidants [18]. In particular, oxidative stress is characterized by an excess of reactive oxygen species (ROS) and reactive nitrogen species (RNS), and reactive radical and non-radical derivatives of oxygen and nitrogen (e.g., H_2_O_2_, superoxide, nitric oxide (NO), peroxynitrite), respectively, as a consequence of aging, infection, disease, toxicity or metabolic disorders [18,19]. Reactive species, especially ROS, are mostly produced in mitochondria and peroxisomes at low physiological levels, where they serve as signaling molecules to regulate biological processes such as transcription, cell cycle, inflammation and aging [20,21,22]. High levels of ROS and RNS instead cause oxidative modifications in the main cellular macromolecules (carbohydrates, lipids, proteins and nucleic acids), which can generate cellular and tissue damage [21]. Oxidative stress is involved in a number of acute and chronic pathological processes including chronic obstructive pulmonary disease, cardiovascular diseases, acute and chronic kidney disease, neurodegenerative diseases, macular degeneration, biliary diseases and cancer [23]. It has also been recognized that pathogenesis of IR, as well as progression and macro- and microvascular complications of T2D, are associated with mitochondrial oxidative stress [19,22]. Notably, pancreatic islets exhibit greater susceptibility towards oxidative and nitrosative stress than other tissues, as a result of their low protein and activity levels (from 1 to 30% of those in the liver) of antioxidant enzymes such as GPx, superoxide dismutase (SOD), glutathione peroxidase, and catalase [24,25].

Under hyperglycemic conditions, in islets and β-cells, there is an overproduction of ROS derived from electron leakage in the mitochondrial respiratory chain and from membrane-bound nicotinamide adenine dinucleotide phosphate (NADPH) oxidases (NOXs); these catalyze the cytosolic NADPH-dependent reduction of molecular oxygen to superoxide, which is subsequently dismutated to H_2_O_2_ by SODs [19,26]. Additionally, the dysregulation of endothelial nitric oxide synthase (eNOS), an enzyme with vascular-protective and anti-inflammatory properties due to the production of NO, is among the major sources of ROS [27]. The oxidation of tetrahydrobiopterin, the cofactor of eNOS, is responsible for the increased formation of oxygen-derived radicals due to eNOS uncoupling, contributing to vascular dysfunction associated with T2D [28]; meanwhile, the reaction of NO with superoxide generates peroxynitrite, which is considered a mediator of cytokine-induced islet β cell destruction [29] (Table 1).

**Figure 1:**
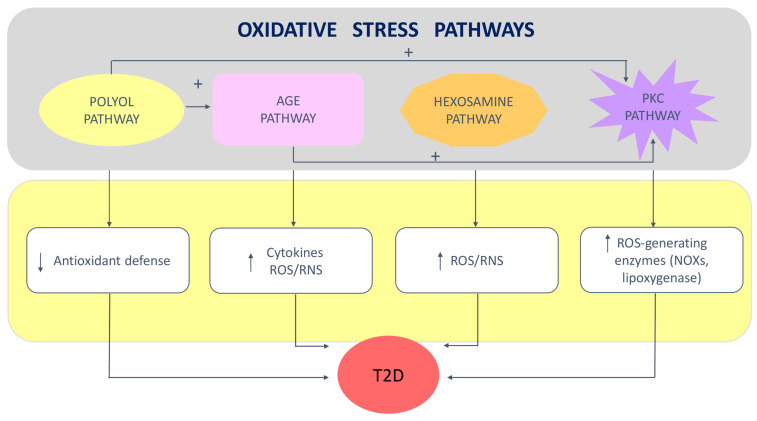
Key oxidative stress pathways in type 2 diabetes: Upper panel: some of these pathways are interconnected and promote other mechanisms generating oxidative stress; Lower panel: arrows represent an increase or a decrease in oxidative stress/inflammation-related biomarkers. Abbreviations: AGE—advanced glycation end product; NOX—NAPDH oxidase; PKC—protein kinase C; RNS—reactive nitrogen species ROS—reactive oxygen species; T2D—type 2 diabetes.

The excess of reactive species due to glucose overload, in turn, induces the activation of four important molecular pathways implicated in the aggravation of T2D [19,23,27,30] (Figure 1): (1) the increased polyol pathway flux depletes NAD(P)H concentration, leading to a reduction in the levels of the antioxidant glutathione (GSH) and in the synthesis of NO; (2) the activation of the advanced glycation end-products (AGEs) pathway occurs through the non-enzymatic reaction of glucose with free amino-acid groups of proteins; AGEs bind to and activate AGE receptors, which then initiate the production of growth factors, proinflammatory cytokines and ROS/RNS; (3) the increase in mitochondrial superoxide concentration results in the enhancement of the hexosamine pathway, which is due the increased levels of fructose-6-phosphate that are moved out from glycolysis, representing an additional source of ROS/RNS; (4) the de novo synthesis of diacylglycerol from glucose, and the greater number of AGEs and their cell receptors, are responsible for the augmented activity of protein kinase C (PKC) isoforms, which is a crucial event in the progression of T2D. In particular, PKC acts at multiple levels by activating eNOS, NOXs, lipoxygenase, phospholipase A2, endothelin-1, vascular endothelial growth factor, transforming growth factor-β, and nuclear factor kappa B; this gives rise to vasoconstriction and the proliferation of vascular muscular cells, increased synthesis of extracellular matrix proteins, and ROS/RNS production mediated by NOXs (reviewed in [23]).

In addition to the decrease in reduced glutathione (GSH) levels, the ratio between GSH and oxidized glutathione, the activity of γ-glutamylcysteine synthase (the rate-limiting enzyme in the synthesis of GSH) [31], and total antioxidant status, an elevation of oxidative markers of DNA damage (8-hydroxy-20-deoxyguanosine), lipid peroxidation (malondialdehyde, 8-isoprostane) and protein oxidation (nitrotyrosine and protein carbonyl levels) have been found in prediabetic and diabetic patients [19,32,33,34]. Furthermore, the Keap1 (Kelch-like ECH-associated protein 1) nuclear factor erythroid 2-related factor-2 (Nrf2) plays important roles in the oxidative stress response [35]. Meanwhile, in non-stress conditions, the transcription factor Nrf2, known as the “master regulator” of the antioxidant response, as it controls the expression of genes involved in the detoxification and elimination of reactive oxidants, is retained in the cytoplasm by the negative regulator Keap1; in response to oxidative stress, it moves to the nucleus where it enhances the expression of various genes implicated in the antioxidant response [33,36,37]. In animal models, the induction of Nrf2 prevents reactive species damage [35], improves insulin sensitivity [38], and protects from diabetes complications [39]. Conversely, low levels of Nrf2 were observed in prediabetic and diabetic subjects, suggesting an impairment of Nrf2 response [27,33] (Table 1).

A pivotal feature of T2D is IR, which verifies when cells are unable to respond properly to insulin [40]. Although low levels of reactive species, namely H_2_O_2_, function as a second messenger in the early insulin-signaling cascade, an excess of H_2_O_2_ results in the continued downregulation of glucose transporter 4 (GLUT4)—the insulin-sensitive glucose transporter that provides insulin-stimulated glucose uptake by adipose tissue and skeletal muscle—and finally, in IR [41,42]. In the normal pathway, the insulin binding with the insulin tyrosine kinase receptor triggers a consecutive phosphorylation of insulin receptor substrate-1 (IRS-1), phosphatidylinositol-3,4,5-triphosphate kinase (PI3K), phosphatidylinositol-4,5-biphosphate kinase, and protein kinase B (Akt), resulting in GLUT4 translocation to the plasma membrane to allow the extracellular glucose to enter peripheral cells [22]. Above the optimal insulin concentration, a shift in the signaling pathway at PI3K induces IR through the serine phosphorylation of IRS proteins, thereby transporting GLUT4 to lysosomes for degradation; consequently, this leads to hyperglycemia, hyperinsulinemia, and increased oxidative stress in tissues [43]. Furthermore, increased H_2_O_2_ levels due to NOX 4-mediated superoxide production stimulate nuclear factor κB (NF-κB), c-Jun N-terminal kinase and p38 mitogen-activated protein kinases (MAPKs), in turn, causing a mitochondria-induced stress response [22]. ROS production is, therefore, crucial in the development of both mitochondrial dysfunction and IR. Indeed, a high intake of glucose provides numerous substrates used by mitochondria for the production of a large amount of ATP, making the mitochondria hyperactive and leading to the overproduction of free radicals [23]. Elevated free-radical species are also responsible for altered mitochondrial dynamics, further contributing to mitochondrial dysfunction [44]. In particular, the increased mitochondrial fission machinery in the skeletal muscle leads to IR and can be rescued through inhibiting fission, which decreases the activity of p38 MAPK and increases IRS-1 and Akt activation, finally resulting in improved muscle insulin signaling and systemic insulin sensitivity [44]. In addition, alterations in mitochondrial function have been shown to be directly involved in the pathogenesis of IR, since primary defects in mitochondrial fatty-acid oxidation capacity cause diacylglycerol accumulation, PKC-epsilon activation, and hepatic IR [45].

Chronic hyperglycemia and the subsequent induction of oxidative stress further suppress nuclear expression of the key β-cell transcription factors PDX-1 and MAFA, leading to a decrease in insulin synthesis and secretion, a process known as β-cell glucose toxicity [46] (Table 1).

## 3. The Relationship between Selenium and Type 2 Diabetes

Selenium is acknowledged as one of the most antioxidant nutrients in humans [47]. Its biological role is principally mediated by Sec that is incorporated into 25 Se-containing proteins including [48]: the ROS-detoxifying selenoenzyme GPx (types 1–7), thioredoxin reductase (TrxR, types 1–3), and methionine sulfoxide reductase B, which take part in the cellular mechanisms against oxidative stress [41,49]; iodothyronine deiodinases (DIO1, DIO2 and DIO3), which maintain the metabolism of THs [50]; selenoprotein P (SELENOP), a protein that primarily serves as a Se transporter to target tissues for the synthesis of extracellular selenoproteins [51]; selenoprotein S (SELENOS), with roles in protein folding and inflammation in the endoplasmic reticulum (ER) [52]; and selenophosphate synthetase, involved in Sec synthesis [53]. GPx and TrxR can both reduce H_2_O_2_ and organic hydroperoxides, while SELENOP, which is mostly secreted by the liver and expressed at relatively high levels in pancreatic islets, also fulfills an antioxidant function in β-cells, displaying both phospholipid hydroperoxide GPx-like and peroxynitrite reductase-like activities [41,54].

The main source of Se is the diet in which the element is mainly contained as Sec and selenomethionine and, at a lower concentration, as the inorganic compounds selenate and selenite [55]. Selenium amounts in plant and animal foods show great variability because of geographical variations in Se content and bioavailability of species in soil and water, the use of fertilizers, and Se supplementation [56,57]. In fact, while certain dry regions of the United States, Canada, South America, China and Russia are characterized by so-called seleniferous soils, other regions such as Finland, Sweden and Scotland are Se deficient [56]. As the extent of population intake is more variable for Se than for other nutrients, human Se status reflects these wide differences [12].

**Figure 2:**
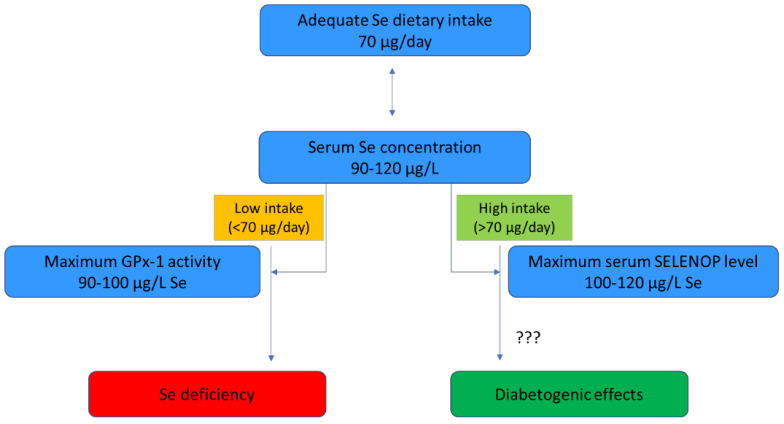
The range of adequate Se dietary intake and its relationship with serum Se levels, the concentration of serum Se biomarkers and potential diabetogenic effects. Abbreviations: GPx-1—glutathione peroxidase 1; Se—selenium; SELENOP—selenoprotein P.

The daily adequate intake for Se has been established at 70 µg by the European Food Safety Authority in the European Union [55]. GPx activity is known to accurately reflect Se status, although GPx1 activity achieves a plateau at a relatively low plasma- or serum-Se concentration (90–100 µg/L) [58]. Therefore, platelet GPx activity is preferably used in populations with a low Se status at baseline [59]. SELENOP accounts for 50% of Se in the blood and is considered a more informative biomarker in populations with relatively low-to-moderate Se intakes [59]. Unlike GPx1, the optimization of SELENOP requires a relatively high Se intake of 100 to 120 µg/L; thus, its concentration reflects the saturation of the functional Se body pool, in order to meet the Se requirement [55,60,61]. Notably, the narrow range (90–120 µg/L) of adequate serum Se makes a key feature of Se status the dependence on a subtle balance between meeting Se nutritional requirements and avoiding excessive exposure [62] (Figure 2).

### 3.1. Experimental Studies

Experimental evidence suggested a direct influence of Se and selenoproteins on the function of pancreatic β-cells [41]. Due to its antioxidant properties, Se has long been believed to prevent T2D by counteracting oxidative stress [63]. Indeed, early studies indicated that high Se doses acted as an insulin mimic, stimulating glucose uptake in isolated rat adipocytes and improving glucose homeostasis in diabetic mice (reviewed in [64]). In addition, selenoprotein deficiency in mice was shown to be associated with T2D and metabolic syndrome (MetS) [64]. Following dietary Se restriction, mice lacking selenocysteine lyase (Scly), the enzyme that provides Se for selenoprotein biosynthesis, developed hyperinsulinemia, glucose intolerance and hepatic steatosis, along with increased hepatic oxidative stress, demonstrating a dependence of glucose and lipid homeostasis on Scly activity [65].

More recently, inorganic and organic Se compounds were reported to stimulate insulin biosynthesis and secretion, probably through upregulation of GPx1 which, on the one hand, protects β-cells from the adverse effects of chronic hyperglycemia (i.e., oxidative stress) [66]; on the other hand, it could lead to hyperinsulinemia and IR [67,68,69]. In particular, higher GPx1 activity was associated with increased activity of protein tyrosine phosphatase 1B that, by dephosphorylating the insulin receptor and the IRS-1, suppresses insulin-induced signaling [70,71]. Conversely, the overproduction of SELENOP contributed to the development of IR and hyperglycemia in liver and skeletal muscle, whereas SELENOP knock-out mice fed a high-sucrose diet were protected against glucose intolerance and IR [72]. Compared to previous studies, these experiments have been carried out in normal but not in diabetic animals using Se levels not exceeding the maximum tolerated dose, and for a longer treatment period [64] (Figure 2).

### 3.2. Epidemiological Studies

A series of non-experimental studies have explored the potential role of Se in the development of T2D in humans, albeit with mixed results, probably as a consequence of different Se status and intake in various world regions, as well as in discrepancies in the absorption rates of different Se forms [47,63]. Various case–control studies, in particular, revealed that serum Se levels were significantly lower in T2D patients than in healthy individuals due to the effect of disease and its related inflammation [73,74,75,76,77].

**Figure 3:**
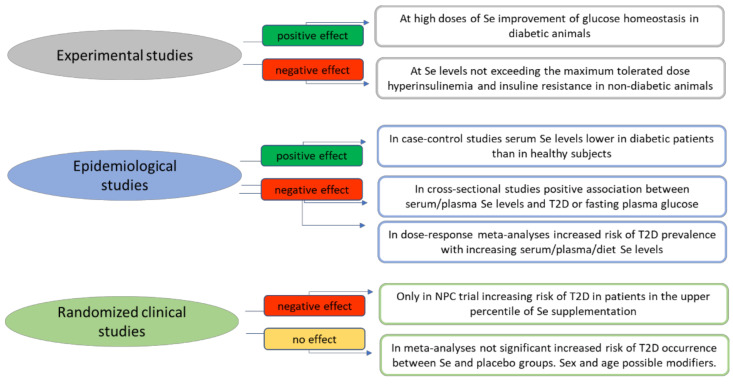
Summary of the current evidence derived from experimental, epidemiological and randomized clinical studies on the association between selenium and occurrence of type 2 diabetes. Abbreviations: NPC—Nutritional Prevention Cancer; T2D—type 2 diabetes.

Some systematic reviews and meta-analyses have also recently been published. Rayman and Stranges [12] reported that, among eleven studies evaluating this relationship, five of them found a significantly positive association between serum/plasma Se and T2D or fasting plasma-glucose; however, their cross-sectional design does not allow the inference of causality, nor can it rule out reverse causation. On the other hand, longitudinal cohort studies did not support this association [12]. A total of five observational studies were included in a non-linear dose–response meta-analysis by Wang and co-authors [47]; it showed a significantly higher prevalence of T2D in the highest category of blood Se compared with the lowest, with an odds ratio (OR) (95% confidence interval, CI) of 1.63 (1.04–2.56, P = 0.033). A substantial degree of heterogeneity across studies, but no evidence of publication bias, was found. Additionally, an increased prevalence of T2D was seen at serum Se concentrations above 132.5 μg/L and below 97.5 μg/L, but not at the middle level (97.5–132.5), suggesting a U-shaped association between Se and T2D [47]. Vinceti et al. [78] performed a dose–response meta-analysis including 50 observational studies (18 cross-sectional, 25 case–control, and 7 cohort studies). They found an increase in the risk of T2D for increasing serum or plasma-Se concentration, with an RR of 3.6 (95% CI = 1.4–9.4) when Se exposure at 140 μg/L was compared with the referent category at 45 μg/L. Additionally, consistently with [47], in these studies, no significant evidence of publication bias was detected. A subsequent systematic review and meta-analysis including a total of thirteen studies (three longitudinal, five case–control and five cross-sectional) demonstrated a significantly positive association between Se concentration and the prevalence of T2D, with a pooled OR of 2.03 (95% CI = 1.51–2.72) [62]. Nevertheless, considerable heterogeneity and a tendency towards publication bias was observed between studies. A total of twenty studies (17 cross-sectional, 3 cohort) were included in the meta-analysis by Kim et al. [13] who, in accordance with previous publications, showed a significantly direct association between Se exposure and the prevalence of T2D (pooled OR = 1.88, 95% CI = 1.44–2.45). Such results were corroborated by subgroup analyses based on Se measurements in blood, urine and diet (but not in nails), sensitivity analysis, and trim and fill analysis for publication bias [13]. In 2021, Vinceti and co-workers conducted an update of their precedent meta-analysis, and retrieved 34 observational studies (18 cross-sectional, 9 cohort, 7 case–control) that measured Se in serum, plasma, whole blood, nail and urine, and from dietary assessment [79]. Compared with the reference blood-Se level category of 90 µg/L, levels of 120 and 160 µg/L were associated with RRs of 1.27 (95% CI = 1.10–1.47) and 1.96 (95% CI = 1.27–3.03), respectively. As for Se from the diet, an intake of 80 µg/day was associated with an RR of 1.23 (95% CI = 1.14–1.33) when compared to the reference category of 55 µg/day, displaying a non-linear relationship in contrast to the J-shaped curved observed for blood-Se levels [79] (Figure 3). The association between increasing urinary Se concentration and T2D did not reach statistical significance, whereas studies based on nail Se indicated an inverted U-shaped association with no significantly decreased RRs at increasing Se concentrations, in contrast with the other results. Unlike other biological matrices, nail Se is not considered a reliable biomarker, as it cannot adequately reflect dietary intake; this positively correlates with organic Se species, mainly SELENOP and Sec, and inversely with inorganic forms (selenate and selenite) [80]. On the other hand, the meta-analysis based on dietary Se intake suggests a threshold of 60–80 µg/day at which the diabetogenic activity of Se could occur [79]. This finding is consistent with both the study of Gu et al. [81], which reported an association between dietary Se intake exceeding 60 μg/day and IR, and the recommended adequate levels of dietary Se intake [55] (Figure 2).

### 3.3. Randomized Clinical Trials

Several randomized clinical trials (RCTs) have also investigated the effects of Se on the risk of T2D development. In the five RCTs included in [12], T2D was considered as a secondary outcome. Only in the NPC (Nutritional Prevention Cancer) trial, in which 1312 subjects with a history of nonmelanoma skin cancer were given 200 µg/day of Se as high-Se yeast or placebo yeast, an increased risk of developing T2D was observed in those patients in the top tertile of Se status at baseline (>122 µg/L) (hazard ratio, HR = 2.70, 95% CI = 1.30–5.61) [82]. This result was neither confirmed in the Selenium and Vitamin E Cancer Prevention Trial (SELECT) nor in the other trials reviewed, despite the common high Se status at baseline in some of them. Regarding the other trials, they were based on a small sample size (Watchful Waiting Trial and Hypocaloric Diet Enriched in Legumes (HDEL) and selenium trial [83,84]), enrolled only male participants (SELECT [85,86]), had a short duration and a restricted age range of participants (PRECISE trial [87]), and did not measure Se at baseline (HDEL and selenium trial [84]). In a subsequent meta-analysis including three RCTs (NCP, SELECT and Sel/Cel trials), which provided participants (a total of 20,290 subjects) with 200 µg/day of Se supplement as either selenized yeast or selenomethionine, no statistically significant increased risk of T2D (pooled OR = 1.18, 95% CI = 0.95–1.47) was found by comparing those who received Se and placebo group [62]. As highlighted by Rayman and Stranges [12], SELECT used selenomethionine, which, despite being the main Se species in food, must undergo catabolism before being used for the synthesis of selenoproteins. Moreover, in the Sel/Cel trial, which administered selenized yeast as in the NPC trial, there was an increased risk for T2D (HR  =  2.21, 95% CI  =  1.04–4.67) only in those individuals aged 63 years or older who had received the supplement, but not on overall participants [88]. The meta-analysis by Vinceti and co-authors [78] retrieved five RCT, all of which administered 200 µg/day to the intervention group, as Se yeast (four studies) or selenomethionine (one study). The pooled relative risk (RR) including all subjects (11,469 and 10,796 subjects in the treatment and placebo group, respectively) was 1.11 (95% CI = 1.01–1.25). Limiting the analysis by sex, a summary RR = 1.10 (95% CI = 1.00–1.24, four studies) was obtained in men and a non-significant estimate in women (RR = 1.43, 95% CI = 0.74–2.77, two studies) [78]. Hence, both age and sex could act as modifiers in the relationship between Se and T2D occurrence [78] (Figure 2).

Evidence from clinical trials does not exclude a potential role of Se supplementation in T2D development, although risk estimates are lower than those from observational studies [12,62]. In addition to the factors already considered, a further explanation for this discrepancy lies in the non-linear association between Se exposure and T2D risk for Se concentrations above 140 µg/L and, since plasma-Se levels (sum baseline plus supplement) were higher in experimental human studies than those in non-experimental studies, a lack of increase or even a decrease in the excess risk for T2D might occur at high Se concentrations [78]. Indeed, most of the RCTs performed so far have been from the United States, where the average dietary intake of Se ranges from 93 µg/day in women to 134 µg/day in men, values substantially higher than those recorded in Europe (40 µg/day) [89]. Furthermore, unlike observational studies that generally cover a longer-term period, Se supplementation in RCTs has a median duration of 3–5 years among older participants, which could be an insufficient period to detect an association of risk [62]. Of note, none of the RCTs conducted employed inorganic Se forms, which are expected to have more pronounced diabetogenic action, consistent with their greater toxicity [90].

### 3.4. Molecular Mechanisms Underlying the Association between Selenium Exposure and Type 2 Diabetes

The relationship between Se and carbohydrate metabolism could be explained by considering that the biosynthesis and secretion of SELENOP are increased under hyperglycemic conditions [71]. SELENOP transcription is controlled by a binding motif in the promoter for FoxO1a and HNF-4α transcription factors, which are co-activated by PGC-1α; these are all known to be involved in the transcriptional regulation of fundamental gluconeogenic enzymes such as glucose-6-phosphatase and phosphoenolpyruvate carboxykinase [41,71]. This finding suggests that SELENOP may also be regulated in hepatocytes such as a gluconeogenic enzyme, with insulin inhibiting gene expression via Akt-mediated phosphorylation of FoxO1a; meanwhile, high glucose stimulates the transcription of gluconeogenic enzymes through post-translational modification of FoxO1a and increasing expression of PGC-1α [41]. Furthermore, the regulation of SELENOP expression in hepatocytes relies on S-adenosylmethionine-dependent protein methylation in a manner similar to that of the gluconeogenic enzymes [91]. Indeed, insulin-resistant individuals with nonalcoholic steatohepatitis displayed a lower rate of transmethylation of methionine [92], while both expression and serum SELENOP concentration were higher in patients with dysregulation of glucose metabolism (T2D or pre-T2D) than those with normal glucose tolerance [72,93,94]. It is important to note, however, that serum SELENOP levels reach saturation with a high Se intake and, as Se deficiency has been reported in patients with T2D, the elevation of SELENOP could be an effect of disease status, rather than the cause [63]. The metabolic actions of SELENOP in the liver—impairment of insulin signaling and dysregulation of glucose metabolism—are mediated, at least partly, by the inactivation of adenosinemonophosphate-activated protein kinase (AMPK), a crucial enzyme that, in physiological conditions, phosphorylates and activates the insulin receptor and promotes activation of the insulin-signaling pathway [72,95]. Genetic variation in SELENOP has been reported to be related to several metabolic phenotypes and, in particular, specific variants were associated with fasting insulin levels and acute insulin response [96]. Finally, a recent study showed that the elevation of circulating SELENOP, but not of circulating Se, was connected to the future onset of hyperglycemia, probably due to the direct anti-oxidative enzyme activity of SELENOP independent of its Se transport capacity [97].

## 4. Thyroid and Type 2 Diabetes

Thyroid diseases and T2D often coexist in the same patients, reinforcing the hypothesis of a reciprocal relationship between these two disorders [9,98,99,100]. Indeed, THs exert a series of effects on glucose metabolism, as suggested by the action of THs on the pancreas in modulating the development and function of pancreatic β-cells [101,102]. In particular, experimental data suggest that triiodothyronine (T3) may induce pancreatic β-cell proliferation in a time-dependent manner via the MAPK/ERK pathway [103]. THs may also affect insulin secretion and glucose uptake in other organs, including the gastrointestinal system (increased glucose absorption), liver (increase in gluconeogenesis and glycogenolysis), skeletal muscle (increased glucose uptake), and adipose tissue (increased lipolysis) [104]. Furthermore, T3 can exert central effects through critical intrahypothalamic actions, with a significant impact on hepatic glucose-production and insulin sensitivity [105]; moreover, it regulates the expression of genes involved in hepatic metabolic responses such as gluconeogenesis, lipogenesis, insulin signaling, adenylate cyclase signaling, cell proliferation, and apoptosis [106,107]. THs may also indirectly lead to T2D complications, as they are positively associated with central adiposity and cardiometabolic risk factors, including increased blood pressure and dyslipidemia [100,108].

Both hypothyroidism and hyperthyroidism have been linked with IR and T2D, with a high prevalence of thyroid dysfunction—up to 29% in T2D patients—and with females more affected than males [109,110]. In particular, the prevalence of thyroid disease appeared to increase with age and in patients with poor glycemic control, while diabetic subjects with thyroid dysfunction displayed dyslipidemia when compared to diabetic patients with normal thyroid function [109]. Untreated hyperthyroid patients (Graves’ disease) exhibited typical signs of β-cell dysfunction, namely the inability to adequately increase the insulin response to hyperglycemia, as well as an augmentation of proinsulin levels both in the fasting state and following a meal [111]. Additionally, the fasting glucose and free fatty-acid levels, as well as insulin concentration and lipid oxidation, were significantly higher in thyrotoxic patients than in controls [111]. In thyrotoxicosis, T3 levels were positively correlated with IR, with the glucagon-to-ghrelin ratio identified as a significant determinant in this relationship [112]. Although high levels of T3 may cause IR in muscle and adipose tissue, only recently, a study documented that high T3 levels are further capable of inducing IR in β-cells by activating endoplasmic reticulum stress (ERS) (increased expression of ERS-related proteins PERK, IRE1, ATF6 and GRP78) and the apoptotic pathway (upregulation of the ERS apoptosis markers CHOP and caspase-12) [113]. An increased degradation of insulin has also been described in hyperthyroidism, which also determined an increased rate of gluconeogenesis and glycogenolysis, increased glucose uptake in peripheral tissues (especially in skeletal muscle), and the production of proinflammatory cytokines (e.g., interleukin 6 and tumor necrosis factor alpha) from adipose tissue [114].

Importantly, hypothyroidism, both overt and subclinical disease, was also associated with a deterioration of glucose metabolism and elevated whole-body IR; moreover, it appeared to be subject to an increased risk of hypoglycemia as a consequence of the mismatch between insulin and glycemic levels [115,116]. Furthermore, insulin-stimulated glucose transport was reported to decrease in patients with hypothyroidism due to disrupted translocation of GLUT4 on the plasma membrane [117]. On the other hand, IR was correlated with a higher prevalence and larger size of thyroid nodules and increased thyroid volume [118,119].

An association between THs and diabetes has also been demonstrated in pregnant women with gestational diabetes or pre-existing T2D, with possible adverse effects for both maternal and fetal health [120,121]. The relationship between IR and/or T2D, obesity, and thyroid cancer has been recently explored and, although the etiology of such an association is unclear, it may involve high insulin levels, increased body fat, hyperglycemia, and anti-diabetes medications including exogenous insulin use [122]. As for anti-diabetes drugs, the role of metformin in thyroid cancer remains controversial due to its growth-inhibitory effects, which are implicated in AMPK-related pathways, the mammalian target of NF-κB, rapamycin and mitochondrial glycerophosphate dehydrogenase [123]. In contrast, regular human insulin and insulin glargine, at high concentrations, significantly induced thyroid cell proliferation and tumor cell migration in in vitro models [124]. The increase in glucose uptake across the plasma membrane, which is necessary to support energy needs for cancer development, was reported in thyroid cancer cells, in which an elevated expression of GLUT1 and GLUT3 could be mainly involved in the oncogenesis process [125]. Additionally, hyperglycemia can affect cancer cellular growth and proliferation via many other mechanisms, such as increasing oxidative stress and inflammatory cytokines, the protection of cancer cells from apoptosis, the stimulation of cell motility, and adhesion [122].

## 5. Selenoprotein Effects in Thyroid Health and Type 2 Diabetes

Se content is the highest in the thyroid, when considering the amount of Se per gram of tissue, and is necessary for antioxidant function and for the synthesis of THs. Indeed, this element is essential for the production of DIOs—which are responsible for the conversion of thyroxine (T4) to T3, the biologically active form of THs—as well as the conversion of T4 to reverse T3 (rT3), the inactive metabolite of THs. Consequently, a deficiency in Se results in reduced expression and activity of these enzymes, leading to an increase in T4 and a decrease in T3 concentrations (Table 2). Selenoproteins such as those belonging to the GPx and TrxR families—which are implicated in the antioxidant defense system and in redox control in general—have a critical role in the thyroid gland, protecting thyrocytes from harmful consequences due to H_2_O_2_ and ROS, which are generated during the normal synthesis of THs by the follicles. Additionally, they are significant for thyroid homeostasis is SELENOP, the main transporter of Se in the blood (Table 2). Moreover, other selenoproteins (S, K and V) have been identified whose functions are not yet fully characterized, although they are involved in redox regulation processes and the ERS response, inflammation, calcium (Ca) regulation, and the maintenance of membrane-associated multiprotein complexes; moreover, they are also important for the functioning of the thyroid [52,126,127,128].

Notably, these selenoproteins are involved in the onset of T2D and the risk of T2D-associated complications (Table 2). While it is well established that THs are essential for metabolic regulation, including basal metabolic rate [158], the relationship between TH and T2D has been further supported by studies evaluating DIO1 and DIO2 polymorphisms (Table 2). In particular, rs7527713 genetic variants in Dio1 were found to be associated with IR [144], whilst polymorphisms in the Dio2 gene were related to greater IR in diabetic subjects (i.e., rs225017-T/A) and increased risk of T2D (i.e., Thr92Ala) [159,160]. On the other hand, Dio2 is modulated by glucocorticoids and insulin in the brown adipocytes of rats; thus, insulin depletion resulted in a general decrease in DIO2 activity, suggesting a reciprocal crosstalk between glucose homeostasis and thyroid function [161]. Furthermore, cardiac dysfunction in diabetic rats was associated with tissue hypothyroidism, evidenced by an increase in DIO3 protein expression and a trend towards increased tissue Dio3 mRNA expression in untreated diabetic hearts, as well as by improved cardiac pathology following low-dose T3 replacement [132].

Excessive GPX1 activity may be detrimental to T2D and obesity, as demonstrated by the positive association between the overexpression of GPx1 and the impaired glucose clearance, hyperinsulinemia, hyperglycemia, and reduced insulin signaling in a mouse model [162]. Conversely, mice lacking GPx1 showed hypotrophy of islet β-cells, insulin hyposecretion, decreased plasma-insulin concentration, and increased insulin sensitivity due to enhanced insulin signaling [163,164]. A number of single-nucleotide polymorphisms (SNPs) in the GPx1 gene have been associated with obesity, a higher prevalence of MetS, IR, and susceptibility to T2D [135,165,166]. As it concerns GPx3, its serum levels were higher in individuals with MetS compared to controls, and GPx3 gene polymorphism (rs8177409) was related to cardiovascular risk in a Mexican population [137]. In contrast, serum GPx3 activity was significantly and inversely correlated to mean carotid intima-media thickness and carotid plaque (indexes of atherosclerosis) in a cohort of patients with T2D, suggesting that both excess and deficiency of GPx3 may cause deleterious effects [139]. SNPs of Gpx3 (rs2230303 and rs8177413) were associated with an increased risk of diabetic kidney disease, the most common T2D complication and the major cause of end-stage kidney disease [138]. Gpx4 haploinsufficient (GPx4+/−) mice fed diet-induced obesity exhibited glucose intolerance, dyslipidemia, cardiac hypertrophy and fibrosis as a consequence of significant increases in lipid peroxide-derived aldehydes [167]. Furthermore, biochemical analysis performed on samples of human atrial myocardium revealed that subjects with T2D and hyperglycemia had significantly decreased GPx4 levels and a greater content of 4-hydroxynonenal (a product of lipid peroxidation) adducts in their heart compared to age-matched non-diabetic patients [167]. Accordingly, lower GPx4 levels were reported in Chinese patients with gestational diabetes than in control pregnant women, with GPx4 negatively correlated with the fasting 2 h plasma-glucose level and glycated albumin in the second trimester [142]. GPX4 expression was also downregulated in high-glucose-stimulated cells, whereas the upregulation of GPX4 expression inhibited ferroptosis (an iron-dependent type of programmed cell death, characterized by iron accumulation and lipid peroxidation), and was able to reduce complex hydroperoxides (e.g., phospholipid hydroperoxides and cholesterol hydroperoxides), breaking the lipid peroxidation chain reaction [143].

The Trx/TrxR system exerts crucial functions in adipocyte dysfunction and obesity, carbohydrate metabolism—including insulin production and sensitivity—β-cell death, blood pressure regulation, inflammation, chemotactic activity of macrophages, and atherogenesis, enough to be proposed as a new additive biomarker for the MetS, IR, and T2D, as well as for the treatment of hypertension and atherosclerosis [147]. In vitro models showed that Trx1 was released by MIN6 insulinoma cells during hypoxic conditions and by pancreatic islet grafts upon glucose stimulation, whereas exogenous supplementation of recombinant Trx1 counteracted apoptosis induced by hypoxia and preserved insulin secretion; this indicates that Trx1 activity supports β-cell function and survival in response to oxidative stressors [145,146]. Certain polymorphisms of the mitochondrial TrxR2 gene (i.e., rs4485648 and rs1548357) were also identified as genetic risk factors of T2D complications such as diabetic retinopathy and myocardial infarction [148,149].

SELENOP, which is also involved in antioxidative defense, has been rediscovered as a “hepatokine” capable of leading to IR and hyperglycemia [168]. In fact, high levels of this protein were found to be increased in T2D, adversely affecting pancreatic β cell functionality and inhibiting insulin secretion, which, in turn, results in impaired insulin signaling and promotion of IR [53]. In addition, elevated plasma-SELENOP levels may predict the onset of glucose intolerance in healthy subjects [97].

Serum SELENOS, a protein principally secreted by hepatocytes, was associated with T2D and its macrovascular complications (macroangiopathy, a specific form of accelerated atherosclerosis) [153,154]. This protein retains antioxidant and anti-inflammatory properties, and contributes to the maintenance of the morphology and distribution of ER in cells, as well as the regulation of ERS; this suggests its potential in the occurrence and development of T2D [151]. Furthermore, several genetic polymorphisms in the SELENOS gene were related to T2D (rs1384565), serum insulin levels (rs4965373), blood-glucose levels, and homeostasis model assessment of IR (rs4965814) [154,155].

SELENOK is a transmembrane protein localized in the ER which protects cells from ERS-induced apoptosis, and is fundamental for promoting Ca(2+) flux during immune cell activation [169,170]. Of note, in vitro, the expression level of SELENOK, as well as that of DIO2, was down-regulated by about 10% due to high levels glucose [149].

Recent experimental studies have highlighted a role for SELENOV in the regulation of Se metabolism and in the functional expression of selenoproteins, as well as in protection against ROS/RNS-mediated ERS oxidative damage [171,172].

## 6. Molecular Targets to Enhance the Antioxidant Defense against Type 2 Diabetes

A promising therapeutic target against T2D is represented by the Keap1–Nrf2 regulatory pathway, which has a central role in cellular protection against oxidative stress, thus diminishing ROS/RNS levels, which contribute to the deterioration of pancreatic β-cells and the consequent reduced release of insulin [173]. Berberine is an alkaloid with a wide range of pharmacological properties, including the suppression of oxidative stress mediated by the Nrf2 pathway; it ultimately promotes an increase in the cellular level of GSH and SOD and a reduction in ROS species [174].

As for selenoproteins, the body of evidence suggests that the suppression of SELENOP may provide a valuable therapeutic approach to improving glucose metabolism and treating T2D and its vascular complications [175]. As reported in Section 3.4, SELENOP is downregulated by insulin and positively regulated by glucose. The antidiabetic drug metformin phosphorylates and inactivates FoxO3a via activation of AMPK, thereby suppressing SELENOP expression in hepatocytes [176]. Eicosapentaenoic acid, a major component of ω-3 polyunsaturated fatty acids, downregulates SELENOP by inactivating sterol regulatory element-binding protein-1c independently of the AMPK pathway [177]. Additionally, a novel molecular strategy based on the development of neutralizing SELENOP monoclonal antibody AE2 was reported to improve glucose intolerance, insulin secretion and IR in vitro and in vivo [178].

ER-resident selenoproteins such as SELENOS and SELENOK arouse particular interest, as they are supposed to modulate inflammatory processes, oxidative stress, and ERS; these are emerging as critical determinants in T2D settings, and therefore, may act as potential pharmacological targets [179]. Moreover, in a model of SELENOV knockout mice, SELENOV emerged as a new inhibitor of body-fat accumulation, activator of energy expenditure, and regulator of O-GlcNAcylation (a protein associated with various metabolic diseases including T2D and obesity), and as such, is an interesting protein to be further investigated in the future [157] (Table 2).

## 7. Conclusions

Accumulated evidence from experimental and epidemiological studies has suggested the potential of Se as a risk factor for T2D; this is supported by the biological plausibility given by the effects of various Se species on glucose metabolism in animals and humans. The relationship between Se and T2D is extremely complex, as emphasized by inconsistencies found both in clinical trials and in animal models. Although in vivo studies are crucial for elucidating the biological mechanisms and pathways involved, animals are more susceptible to variations in Se supply and selenoprotein activities; therefore, their results have more limited translational value. If experimental studies in humans allow the determination of a causal relationship, those performed in populations with suboptimal Se intake and including a wider range of concentrations of Se supplementation, as well as studies investigating the association between participant genotypes and Se status, are currently missing. With respect to observational studies, the generally high heterogeneity found between studies is a consequence of differences in study design, age, sex ratio, and Se status, as well as in the biomarkers used for Se measurement. Overall, these data indicate that, although adequate Se intake is critical to human health, supplementing Se beyond the recommended daily intake may be associated with a small but possible increased risk of T2D.

THs are essential for the homeostatic control of energy metabolism, and exert a variety of effects on glucose metabolism, IR, and T2D and its complications. Conversely, elevated insulin may affect the prevalence and size of thyroid nodules and thyroid volume, revealing a bidirectional interconnection between the two conditions. Beyond their most studied common determinants, selenoproteins are emerging as additional critical factors for both adequate insulin and TH synthesis; as such, they are simultaneously both essential for thyroid functionality and involved in the pathogenesis and development of T2D. Nevertheless, future studies are warranted, to delve into the association between T2D and thyroid dysfunction; these should explore common molecular and cellular mechanisms that may play a role in both conditions, how Se fits into this relationship, and whether Se supplementation (allowing for full selenoprotein activity and avoiding toxic consequences) may contribute to the prevention and/or therapeutic treatment of these globally widespread conditions.

## Figures and Tables

**Table 1:** List of main molecules involved in the onset of insulin resistance and type 2 diabetes.

Molecule	Acronym	Function	Reference
Nicotinamide adenine dinucleotide phosphate oxidases	NOXs	Production of superoxide; mitochondria-induced stress response	[19,22]
Endothelial nitric oxide synthase	eNOS	Production of nitrix oxide	[27]
Tetrahydrobiopterin	-	Formation of oxygen-derived radicals	[28]
Nitric oxide	NO	Reaction with superoxide which generates peroxynitrite	[29]
Kelch-like ECH-associated protein 1	Keap1	Negative regulator of Nrf2	[33]
Nuclear factor erythroid 2-related factor-2	Nrf2	Control of antioxidant response	[35,36,37,38,39]
Hydrogen peroxide	H_2_O_2_	Downregulation of GLUT4; stimulation of NF-κB, c-Jun N-terminal kinase and p38 MAPKs	[22]
Glucose transporter 4	GLUT4	Insulin-stimulated glucose uptake by adipose tissue and skeletal muscle	[42]
Pancreatic and duodenal homeobox 1	PDX-1	β-cell transcription factor	[46]
MAF BZIP Transcription Factor A	MAFA	β-cell transcription factor	[46]

Abbreviations: GLUT4—glucose transporter 4; MAPKs—mitogen-activated protein kinases; NF-κB—nuclear factor κB.

**Table 2:** Selenoproteins involved in thyroid health and type 2 diabetes.

Selenoprotein (Acronym)	Types/ Isoenzymes	Main Functions	Effects Linked to Thyroid	Reference	Effects Linked to T2D	Reference
Iodothyronine deiodinase (DIO)	DIO1 DIO2 DIO3	T4 to T3 conversion T4 to T3 conversion T4 to rT3 conversion	Conversion of TH Conversion of TH Deactivation of TH	[129] [129] [129]	DIO1 polymorphism (rs7527713) associated with IR DIO2 polymorphism (Thr92Ala) associated with BMI and FBG Increased DIO3 associated with cardiac dysfunction in diabetic heart	[130] [131] [132]
Glutathione peroxidase (GPx)	GPx1	Cytosolic antioxidant	Thyrocyte protection from peroxidative damage	[133,134]	Overexpression of GPx1 associated with T2D-like phenotypes. Human GPX1 polymorphism related to risks of diabetes and obesity	[135]
	GPx2	Extracellular antioxidant	Thyrocyte protection from peroxidative damage	[133,136]	GPx3 involved in MetS, IR and T2D complications	[137,138,139,140]
	GPx3	Membrane phospholipid antioxidant	Antioxidant protecting the membrane, regulation of cellular death	[141]	GPx4 levels associated with clinical outcomes and metabolic abnormalities among patients with gestational diabetes mellitus. GPX4 involvement in high-glucose-induced ferroptosis (programmed cell death dependent on iron)	[142,143]
Thioredoxin reductase (TrxR)	TrxR1 TrxR2	Redox state regulation and antioxidant actions	Thyrocyte protection from peroxidative damage Antioxidant	[133,134,144]	Improvement of survival and function of pancreatic β-cells TRx2 polymorphisms associated with T2D complications	[145,146,147] [148,149]
Selenoprotein P (SELENOP)	-	Selenium transport and storage, antioxidant defense	Selenium supply to thyroid	[150]	Relationship with IR and T2D	[51,97]
Selenoprotein S (SELENOS)	-	Protection against ERS	Protection against ERS and oxidative injury	[151,152]	SELENOS levels associated with T2D and T2D macrovascular complications. SELENOS polymorphisms associated with the risk for developing T2D and macroangiopathy.	[153,154,155]
Selenoprotein K (SELENOK)	-	Quality control within the ER	Protection against ERS and oxidative injury	[152]	SELENOK expression downregulated by glucose	[156]
Selenoprotein V (SELENOV)	-	Modulation of redox processes and ER calcium homeostasis, cell adhesion and angiogenesis	Protection against ERS and oxidative injury	[130]	SELENOV as modulator of body fat accumulation and energy expenditure, and regulator of O-GlcNAcylation (protein associated with various metabolic diseases including T2D and obesity)	[157]

Abbreviations: BMI—body-mass index; ER—endoplasmic reticulum; ERS—endoplasmic reticulum stress; FBG—fasting blood-glucose; IR—insulin resistance; MetS—metabolic syndrome; rT3—reverse triiodothyronine; T2D—type 2 diabetes; T3—triiodothyronine; T4—thyroxine; TH—thyroid hormone.

## Abbreviations

AGE	Advanced glycation end-product
Akt	Protein kinase B
ATP	Adenosine triphosphate
DIO	Deiodinase
eNOS	Endothelial nitric oxide synthase
ER	Endoplasmic reticulum
ERS	Endoplasmic reticulum stress
GSH	Reduced glutathione
GPx	Glutathione peroxidase
IR	Insulin resistance
IRS-1	Insulin receptor substrate-1
Keap1	Kelch-like ECH-associated protein 1
MAPK	Mitogen-activated protein kinase
MetS	Metabolic syndrome
NADPH	Nicotinamide adenine dinucleotide phosphate
NF-κB	Nuclear factor κB
NO	Nitrogen oxide
NOX	NAPDH oxidase
Nrf2	Nuclear factor erythroid 2-related factor-2
PI3K	Phosphatidylinositol-3,4,5-triphosphate kinase
PKC	Protein kinase C
RNS	Reactive nitrogen species
ROS	Reactive oxygen species
rT3	Reverse triiodothyronine
Sec	Selenocysteine
SELENOK	Selenoprotein K
SELENOP	Selenoprotein P
SELENOS	Selenoprotein S
SELENOV	Selenoprotein V
SNP	Single-nucleotide polymorphism
SOD	Superoxide dismutase
T2D	Type 2 diabetes
T3	Triiodothyronine
T4	Thyroxine
TH	Thyroid hormone
TrxR	Thioredoxin reductase
